# Supplementary material for: Curated and harmonised transcriptomics datasets of interstitial lung diseases
Source: Data Brief. 2025 Oct 14;63:112139. doi: 10.1016/j.dib.2025.112139 (PMC12581653; doi:10.1016/j.dib.2025.112139)
Supplement: Supplementary file 1 [file mmc1.zip › Supplementary_material/DNA-microarray/GSE70866/GSE70866_eUTOPIA_Agilent_QC_Report_2023-12-15.tar!/Users/OWNER/AppData/Local/Temp/RtmpAFKb7F/2023-12-15/pca.pdf]

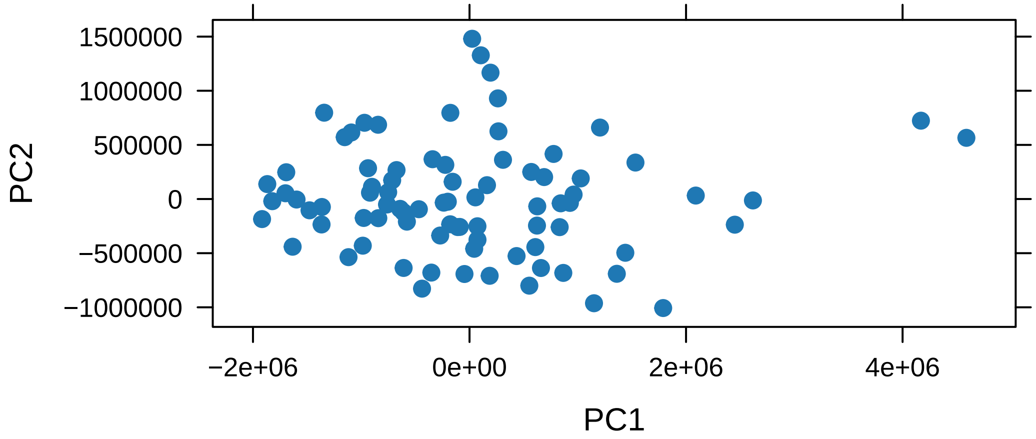

1500000
1000000
500000
0
−500000
−1000000
PC2
−2e+06
0e+00
2e+06
4e+06
PC1
